# The relationship between quality of life and health promotion behavior in patients with type B aortic dissection: a cross-sectional study

**DOI:** 10.1186/s13019-023-02124-5

**Published:** 2023-01-13

**Authors:** Jianxin Tu, Fei Wang, Furong Yin, Linxue Zhang, Benli Zhao, Jiamei Zhou

**Affiliations:** 1 Nursing Department, The Second Affiliated Hospital of Zunyi Medical University, Zunyi, China; 2 Cardiovascular Surgery Department, Affiliated Hospital of Zunyi Medical University, Zunyi, China; 3 Nursing Department, Zunyi Medical University, Zunyi, China; 4 Nursing Department, Affiliated Hospital of Zunyi Medical University, Zunyi, China

**Keywords:** Aortic dissection, Health promotion behavior, Quality of life, Correlation

## Abstract

**Background:**

To understand the current situation of health promotion behavior and quality of life among aortic dissection survivors and the correlation between them.

**Methods:**

Sociodemographic characteristics were collected. T-test and variance analysis were applied for univariate analysis. Quality of life was measured using the SF-36 Questionnaire, and health-promoting behaviors were measured using the aortic dissection health promotion behavior questionnaire. The association between type B aortic dissection survivors’ health promotion behavior and health status questionnaire (SF-36) scores was determined through Pearson’s correlation coefficients. This association was analyzed through multivariable regression analysis.

**Results:**

A total of 131 type B aortic dissection survivors were evaluated through the self-developed aortic dissection patient health promotion behavior scale and health status questionnaire (SF-36). Results showed that the health promotion behavior of Stanford B aortic dissection survivors (85.05 ± 11.28) correlated with their Mental Component Summary (MCS) (55.23 ± 30.72; *r* = 0.359, *P* < 0.01). The model showed 39.00% variance shared between behavior motivation and MCS (*R*^2^ = 0.390, F = 13.189, *P* < 0.01).

**Conclusion:**

Type B aortic dissection survivors in Zunyi, China had a lower quality of life. Medical staff can formulate intervention measures from behavioral motivation to improve the quality of life of aortic dissection survivors.

## Introduction

Aortic dissection (AD) occurs due to a disruption of the aortic-wall integrity and architecture, most often related to underlying genetic disorders, abnormal shear stress, inflammatory disease, trauma, or combinations thereof. According to the Stanford system, type B AD is defined as involving the descending aorta or the arch (the distal to left subclavian artery) without an involvement of the ascending aorta; Stanford B AD accounts for about 20–40% of all AD patients [1]. Currently, there is a 90% survival rate upon hospital discharge for patients with type B AD [1]. Effective disease treatment implies that more patients with AD undergo chronic disease management. However, a study of about 6,937 patients with unplanned admissions for type B AD from 2010 to 2014 reported the following: the nonelective 90-day readmission rate was 25.1%, an additional 4.7% of patients were electively readmitted, the most common cause of nonelective readmission was new or recurrent arterial aneurysm or dissection, the mortality rate during nonelective readmission was 5.0%, and the mean cost of Rehospitalization was from $22,572 to $41,598 [2].

Thoracic endovascular aortic repair (TEVAR) has become the preferred treatment for patients with type B AD. However, patients are at risk of endoleak, continued aneurysm sac expansion, and ultimately endovascular and/or open revision of the thoracic aortic repair following TEVAR [3]. In addition, several studies [4–6] have shown that the quality of life (QoL) of AD survivors is not at a desirable level. The high nonelective readmission rate of type B AD survivors and low QoL level need to be solved urgently.

Health promoting behaviors refer to those behaviors that enable people to improve their own and their society’s health. The areas of health promoting behaviors include nutrition, physical activity, stress management, health responsibilities, interpersonal relationships, and spiritual growth [7]. Health promoting behaviors that have been recommended for patients with AD include dietary control, physical activity, cessation of smoking, medication adherence, and adherence to medical recommendations (including monitoring blood pressure and body weight daily) [8]. These healthy lifestyle interventions are quite effective in reducing the risk of cardiovascular disease while lowering rehospitalization rates [9].

Improving health promoting behaviors may lead to a better QoL and lower incidence of AD events [10]. Most of the past published literature has focused on surgical outcomes, the survival of patients, and complications leading to disability. Postoperative health promoting behaviors and QoL have rarely been considered in this view. Moreover, there is a paucity of data on the multidimensional assessment of health promoting behaviors among type B AD survivors in China, and the relationship between health promoting behaviors and QoL has not been explored. Consequently, it is essential to carry out this investigation to address the gap in research literature.

The present study aimed to identify health promoting behaviors and QoL after TEVAR, focusing on the correlation between health promotion behavior and QoL among survivors on Stanford B AD.

## Methods

### Design

This was a cross-sectional study with data collection through questionnaires.

### Setting

This study was conducted in the cardiovascular surgery of The Affiliated Hospital of Zunyi Medical University.

### Participants

From December 2016 to June 2020, 205 patients received TEVAR for Stanford type B AD. Through convenience sampling, 131 survivors (response rate = 63.9%) agreed to participate in the study. The questionnaire was issued through telephone and was filled out within 30 min. The investigators sorted out and verified the recovered questionnaires on the spot, correcting any missing or wrong questionnaires in time to ensure the quality of questionnaire recovery. Finally, a total of 205 questionnaires were distributed and 131 valid questionnaires were collected.

### Procedure and data collection

The study was conducted from March 2021 to May 2021. Participants were recruited through telephone under the instruction of trained investigators. The questionnaires were completed through telephone inquiry. We obtained every participant’s demographic characteristics, including gender, age, levels of education, and so on.

### Measures

#### Demographic characteristics

Sociodemographic and clinical data were collected from participants using the patients’ files and a self-designed questionnaire, including age, gender, and education level.

#### SF-36 questionnaire

The SF-36 measures eight QoL domains, which are dichotomized into physical (functioning, role limitations (physical), pain, general health) and mental health (vitality, social functioning, role limitations (emotional), and emotional/ mental health). and including 2 main scales, the Mental Component Summary(MCS) and the Physical Component Summary (PCS). Item scores were converted to a 0–100 point scale; domain scores were derived by averaging individual items within the subscale; higher values are indicative of better QoL [11].

#### AD health promotion behavior questionnaire

The AD health promotion behavior questionnaire is a self-designed questionnaire including five domains: nutrition (six items), exercise (six items), blood pressure management (five items), disease knowledge (five items), and behavioral motive (three items). Each item uses a five-point scale to measure the level of agreement with each statement (1 = never, 2 = seldom, 3 = sometimes, 4 = often, 5 = always). A score is calculated for each domain, and the sum of scores corresponds to the level of overall health behavior. Higher scores indicate better health behavior. The Cronbach’s α was 0.691–0.907, the reliability was 0.930, and the validity was 0.796 [12].

### Ethical considerations

This study was conducted according to the ethical guidelines described in the Helsinki Declaration (World Medical Association, 2013). The study was approved by the Affiliated Hospital of Zunyi Medical University (Approval No.: KLLY-2020-014). All subjects provided informed consent to participate in the study. The data underlying this article will be shared upon reasonable request by the corresponding author.

## Data analysis

Descriptive statistics (mean, standard deviation, frequency, and percentage) were used to describe the sample’s characteristics. T-test and variance analysis were applied for univariate analysis, with Pearson’s correlation and multivariable regression being applied for multivariable analysis. Normal distribution and the types of variables were considered to decide which tests were suitable for the analysis. For the continuous variables, such as “age,” patients were stratified into different subgroups based on logical numerical break points. Age was stratified by decades into < 44 years, 45–54 years, 55–64 years, and ≥ 65 years. All data were input in Excel and analyzed with SPSS Statistics for Windows, version 18.0. Significance was assessed at *P* < 0.05 level in the bivariate analysis.

## Results

A total of 131 participants were included in the study with more men (80.15%) than women (19.85%). The ages of these patients ranged from 25 to 86 years. Demographic information for the study sample is presented in Table 1.

**Table 1:** Demographic characteristics of participants (*N* = 131)

Variable	Categories	Mean (SD)	Frequency (N)	Percentage
Gender	Men		105	80.15
	Women		26	19.85
Age (years)	< 44		7	6.60
	45–54		25	21.40
	55–64		39	32.40
	≥ 65		60	39.70
Degree of education	Primary school and below		35	26.72
	Junior high school and technical secondary		86	65.65
	High school and above		10	7.63
Course of disease	< 2 years		16	12.21
	3–4 years		49	31.30
	4–5 years		38	29.00
	> 5 years		28	21.37
PCS		59.81 ± 23.01		
MCS		55.23 ± 30.72		
AD health promotion Behavior questionnaire		85.05 ± 11.28		

*AD* aortic dissection, *MCS* mental component summary, *PCS* physical component summary

As demonstrated in Table 2, the mean total score of health behavior was 85.05 ± 11.28. Disease knowledge showed the lowest score (13.27 ± 3.88) followed by exercise (19.34 ± 4.58) and nutrition (21.05 ± 3.59). As demonstrated in Table 3, compared with the QoL scores of Chinese norm samples, the evaluation showed that the average pain score of type B AD survivors was higher than that of the Chinese norm samples (91.39 ± 13.56 vs 80.40 ± 19.75), and the scores of other dimensions were lower than those of the Chinese norm samples.

**Table 2:** Scores of health promoting behavior in patients with type B AD ($\overline{x}$ ± SD)

Domains	Score of each dimension	Total score of the dimension	Standardized scores	Score ranking
Exercise	19.34 ± 4.58	30	64.47	4
Blood pressure Management	20.64 ± 3.92	25	82.56	1
Disease knowledge	13.27 ± 3.88	25	53.08	5
Nutrition	21.05 ± 3.59	30	70.17	3
Behavioral motive	10.75 ± 2.36	15	71.67	2

Standardized score index = (Score of each dimension/total score of dimension) × 100%

*AD* aortic dissection

**Table 3:** Comparison of QoL scores in patients with type B AD and Chinese norm samples ($\overline{x}$ ± SD)

Domains	Type B AD Scores	Chinese norm samples scores
Physical Functioning	70.99 ± 20.43	89.1 ± 15.73
Role Limitations (physical)	33.97 ± 44.3	81.99 ± 31.65
Pain	91.39 ± 13.56	80.40 ± 19.75
General Health	51.26 ± 18.69	66.03 ± 20.87
Vitality	64.08 ± 19.71	71.15 ± 18.09
Social Functioning	56.20 ± 27.18	84.60 ± 18.15
Mental Health	59.76 ± 20.70	75.23 ± 16.69
Role Limitations (emotional)	63.87 ± 43.76	77.04 ± 35.45

*AD* aortic dissection

Table 4 shows the average item score for each subscale. In terms of physical functioning, there were statistical differences for both age and education; the mean scores of physical functioning continuously decreased with increasing age (*p* < 0.001).

**Table 4:** Associations and differences of QoL mean scores with demographic variables

Characteristic	Physical functioning	Role limitations (physical)	Pain	General health	Vitality	Social functioning	Mental health	Role limitations (emotional)	PCS	MCS
*Gender*										
Men	71.29 ± 20.99	35.24 ± 45.43	92.69 ± 12.17	52.71 ± 18.48	65.24 ± 20.44	58.10 ± 28.11	65.08 ± 43.21	60.46 ± 21.19	60.79 ± 21.94	56.51 ± 31.61
Women	69.81 ± 18.36	28.85 ± 39.81	86.16 ± 17.44	45.38 ± 18.76	59.42 ± 15.90	48.56 ± 21.89	58.97 ± 46.48	56.92 ± 18.70	55.87 ± 27.05	50.10 ± 26.80
P value	0.743	0.512	0.127	0.073	0.175	0.110	0.526	0.538	0.331	0.342
*Age (years)*										
< 44	85.71 ± 3.45	42.86 ± 53.45	94.71 ± 9.03	72.86 ± 14.68	85.00 ± 9.13	78.57 ± 25.73	77.14 ± 19.00	66.67 ± 43.03	71.68 ± 22.59	70.83 ± 33.20
45–54	82.00 ± 9.90	46.00 ± 49.31	92.30 ± 11.21	56.80 ± 17.73	68.20 ± 18.65	72.00 ± 20.50	61.12 ± 22.41	72.00 ± 34.26	68.51 ± 19.79	60.74 ± 25.33
55–64	78.85 ± 8.62	43.59 ± 45.42	94.49 ± 9.35	54.62 ± 15.66	67.82 ± 17.35	58.65 ± 24.86	65.33 ± 19.84	70.94 ± 44.71	65.84 ± 20.20	59.37 ± 33.19
≥ 65	59.58 ± 24.10	21.67 ± 37.81	88.62 ± 16.53	44.25 ± 18.34	57.50 ± 19.99	45.42 ± 26.54	53.53 ± 18.79	55.56 ± 46.20	50.88 ± 23.22	48.43 ± 29.95
*P* value	< 0.001	0.033	0.166	< 0.001	< 0.001	< 0.001	0.003	0.255	< 0.001	0.098
*Degree of education*										
primary school and below	61.43 ± 23.22	23.57 ± 39.27	89.94 ± 15.92	44.57 ± 17.25	58.71 ± 20.70	47.86 ± 26.34	55.31 ± 19.75	56.19 ± 46.98	52.34 ± 23.20	49.73 ± 31.85
junior high school and technical secondary	73.37 ± 18.86	37.50 ± 45.17	91.41 ± 13.02	51.92 ± 18.10	64.01 ± 18.78	56.83 ± 26.48	59.40 ± 20.32	65.89 ± 43.08	61.91 ± 22.54	55.18 ± 29.91
high school and above	84.00 ± 6.15	40.00 ± 51.64	96.30 ± 7.80	69.00 ± 17.29	83.50 ± 11.32	80.00 ± 22.97	78.40 ± 18.78	73.33 ± 37.84	67.88 ± 21.98	74.90 ± 28.37
*P* value	< 0.001	0.266	0.428	< 0.001	0.002	0.003	0.007	0.424	0.059	0.073
*Course of disease*										
< 2 years	62.50 ± 28.17	9.38 ± 17.97	93.38 ± 8.86	56.25 ± 18.21	67.50 ± 16.53	57.81 ± 26.57	73.50 ± 16.52	58.33 ± 44.72	59.83 ± 13.34	64.77 ± 24.98
3–4 years	75.31 ± 14.70	20.41 ± 38.08	91.26 ± 10.89	51.33 ± 19.55	62.96 ± 17.56	50.26 ± 24.93	55.43 ± 18.53	59.18 ± 42.64	58.27 ± 15.82	49.58 ± 28.01
4–5 years	71.58 ± 20.80	50.00 ± 48.63	93.13 ± 13.75	52.11 ± 16.13	70.00 ± 23.37	67.11 ± 29.70	67.58 ± 20.37	78.90 ± 38.3	68.81 ± 21.8	67.98 ± 30.40
> 5 years	67.50 ± 22.42	50.00 ± 46.15	88.14 ± 18.80	47.14 ± 20.70	56.07 ± 17.23	50.90 ± 24.28	48.86 ± 19.42	54.76 ± 49.04	57.92 ± 18.12	42.38 ± 30.31
*P* value	0.121	< 0.001	0.465	0.466	0.032	0.021	< 0.001	0.086	0.073	0.002

The mean scores of physical functioning were higher in high school graduates (84.00 ± 6.15) than in junior high school graduates (73.37 ± 18.86) and primary school graduates (61.43 ± 23.22) (*p* < 0.001). In terms of role limitations (physical), there were statistical differences for both age and course of disease. The mean scores of the patients among 45–54 years old were higher than those of patients among < 44, 55–64 and ≥ 65 years old. The average role limitations (physical) score continued to increase with course of disease (*p* < 0.001).

In terms of general health, there were statistical differences for both age and course of disease; the average general health score continuously decreased with increasing age (*p* < 0.001). The average general health score continued to increase with education (*p* < 0.001). In terms of vitality, there were statistical differences among age, education, and course of disease. The average vitality score continuously decreased with increasing age (*p* < 0.001) and continued to increase with education (*p* = 0.002). Moreover, the mean scores of the patients with 4–5 years’ duration of type B AD were higher than those of patients with < 2, 3–4, and > 5 years’ duration (*p* = 0.032).

In terms of social functioning, there were statistical differences among age, education, and course of disease. The average social functioning score continuously decreased with increasing age (*p* < 0.001) and continued to increase with education (*p* = 0.003); the mean scores of the patients with 4–5 years’ duration of type B AD were higher than those of patients with < 2, 3–4, and > 5 years’ duration (*p* = 0.021).

In terms of mental health, there were statistical differences among age, education, and course of disease. The average mental health score continuously decreased with increasing age (*p* = 0.003) and continued to increase with education (*p* = 0.007); the mean scores of the patients with < 2 years’ duration of type B AD were higher than those of patients with 3–4, 4–5, and > 5 years’ duration (*p* < 0.001). In terms of MCS, the mean scores of the patients with 4–5 years’ duration of type B AD were higher than those of patients with < 2, 3–4, and > 5 years’ duration (*p* = 0.002), and the PCS continuously decreased with increasing age (*p* < 0.001).

The correlation analysis showed that the total score of the health promotion behavior scale was correlated with that of the MCS (r = 0.359, *P* < 0.01); this meant that the higher the level of health promotion behavior, the higher the MCS (Table 5).

**Table 5:** Correlation between health-promoting lifestyle and QoL (*N* = 131, r)

	Scores of health promotion behavior	Behavior motive	Nutrition	Blood pressure management	Disease knowledge	Exercise
PCS	.150	.013	.205*	− .089	.057	.230**
MCS	.359**	.614**	.125	− .003	.212*	.293**
Physical Functioning	.206*	.212*	.190*	− .199*	.063	.367**
Role Limitations (physical)	.202*	.139	.229**	− .056	.168	.150
Pain	.293**	.392**	.115	.069	.076	.308**
General Health	.381**	.454**	.205*	.008	.139	.418**
Vitality	.427**	.576**	.224*	− .067	.186*	.479**
Social Functioning	.129	.156	.044	− .040	.037	.205*
Mental Health	.451**	.668**	.196*	.016	.200*	.430**
Role Limitations (emotional)	.317**	.564**	.139	− .037	.240**	.208*

**At the 0.01 level (two-tailed), the correlation was significant

*At the 0.05 level (two-tailed), the correlation was significant

*AD* aortic dissection

Multiple linear regression analysis for the influencing factors of type B AD survivors was conducted with the total MCS score as the dependent variable. The independent variables were exercise, disease knowledge, behavioral motive, and the statistically significant variables of univariate analysis (entry level was 0.05, deletion level was 0.10). According to the results of the regression equations, behavior motive (B = 7.993, *P* < 0.001) was associated with MCS. The model showed 39.00% variance shared between the dependent and independent variables (F = 13.189, *p* < 0.001) (Table 6).

**Table 6:** Multiple regression analysis of factors influencing AD survivors regarding QoL

Model	Variable	Unstandardized coefficients		Standardized coefficients	t	Sig	95.0% CI for B		R^2^	F	*P* value
		B	SE	Beta			Lower bound	Upper bound			
	Behavior motive	7.993	0.905	0.614	7.682	< 0.001	5.934	10.054	0.390	13.189	< 0.001

*AD* aortic dissection, *SE* standard error, *CI* confidence interval

## Discussion

### The current health promotion behaviors of AD survivors in ZunYi, China

Health promotion behaviors are considered important determinants of health, and promoting them can prevent one-third of the overall mortality; accurate assessment is therefore helpful in identifying the risk of cardiovascular risk factors in AD survivors, preventing and reducing the occurrence of clinical events [9]. At present, the health promotion behavior measurement tools are universal, but the rehabilitation mode, exercise type, and nutritional requirements of AD patients have their particularities. The universal scale is often difficult in terms of reflecting the specificity of AD. Therefore, this study used a self-developed health promotion behavior evaluation scale for AD patients to evaluate their QoL, being more targeted and applicable to Chinese AD patients.

The present study’s findings indicated that ZunYi AD survivors had the lowest scores on their disease knowledge, followed by exercise and nutrition among the five domains. This finding was consistent with Kehler [13] and Thijssen [14]. This may be related to the low incidence of AD compared with other heart diseases, since it is not a well-known disease among the public. AD survivors generally have low rehabilitation knowledge related to AD, resulting in them being unable to identify AD risk factors and physical symptoms. Hence, they are unable to scientifically formulate diet and exercise plans, increasing the risk of recurrence. In addition, due to a lack of disease knowledge, most survivors worry that exercise will increase the risk of AD recurrence. Hence, they reduce or stop exercise, which will further affect their health promotion behavior [12]. Since AD is a chronic disease that requires long-term management, enhancing AD disease knowledge and exercise, including diet programs and health education for patients, may promote AD survivors’ health behavior. In addition, our study found that although most of the patients took medication regularly, nearly 67.2% did not control their blood pressure at the guideline recommended level (100–120/60–80 mmHg). We found that most patients did not know about blood pressure control methods besides drugs, and many did not master the correct method and time of blood pressure measurement, leading to a deviation in blood pressure records. Therefore, in addition to health education of rehabilitation knowledge, training of self-care skills for patients may help prevent the occurrence of long-term complications and reduce the number of hospitalizations.

On the other hand, in our study, most of the participants lived with their family, which may imply that they received more support from family members. With the goal of achieving better behavioral changes for the patient, education should also involve their family as they serve an important role in supporting the patient’s health-promoting behaviors.

### The current QoL situation of type B AD survivors in Zunyi, China

The SF-36 questionnaire has been widely used to evaluate QoL after thoracic aortic surgery [15, 16], including patients with acute type A AD after aortic replacement [17], patients with AD after TEVAR [6], and patients with abdominal aortic aneurysm repair [18]. Therefore, the questionnaire is practical and applicable to the study population.

In total, 131 patients completed the self-report SF-36 questionnaire completely. As demonstrated by the findings (Table 3), the evaluation shows that the average pain score of type B AD survivors was higher than that of the Chinese norm samples (91.39 ± 13.56 vs. 80.40 ± 19.75), and the scores of other dimensions were lower than those of the Chinese norm samples [19]. This may be related to the patient's active control of blood pressure. Compared with the results of type B AD survivors’ survey conducted in European countries, this survey revealed that the type B AD survivors in Zunyi China had a low QoL level [20]. In addition, in this study, the QoL level of type B AD survivors in Zunyi China was much lower than that of type B AD survivors in JiangSu, China, [21]. This may be related to the overall low education level regarding type B AD among the patients selected in this study (92.37% below high school); education level is a positive influencing factor of QoL level.

According to the univariate analysis of QoL scores (Table 3), the lower the education level, the worse the QoL score, which was similar to the study by Zhang [21]. This may be related to the fact that patients with higher education levels are more likely to acquire disease knowledge and have more awareness and understanding of the disease. Additionally, our findings showed that the MCS score of type B AD survivors among 4–5 years after discharge was higher than that at other times, which may be because those with lower QoL died at this point or did not participate in the study. Therefore, nurses should combine China’s medical system and nursing mode, draw lessons from foreign experience to formulate personalized discharge plans for type B AD survivors, pay attention to the discharge readiness of the survivors, and improve their QoL.

### Analysis of the influencing factors of type B AD survivors’ QoL in Zunyi, China

As listed in Table 5, the total scores of health promotion behavior, behavioral motivation, disease knowledge, exercise dimension, and MCS of type B AD survivors were positively correlated (r = 0.359, *P* < 0.01). Further multivariable analysis showed that behavioral motive was the influencing factor of MCS (Table 5).

Behavioral motive is an important factor affecting the QoL of AD survivors. Similar to the research results of Archer [22], behavioral motive can mobilize the rehabilitation enthusiasm of patients to a great extent. In this study, the behavioral motive score of AD survivors was high, indicating that they had high enthusiasm and initiative regarding rehabilitation. Zunyi is a city with relatively low economic levels in China. Thus, most type B AD survivors in Zunyi have limited economic conditions. Out of concern about high medical expenses in cardiac rehabilitation centers, in Chinese institutions, patients after TEVAR are usually not involved in cardiac rehabilitation. Therefore, type B AD survivors in Zunyi have stronger initiatives regarding out-of-hospital active rehabilitation. In view of the strong behavioral motivation of type B AD survivors observed in this study, using behavioral motivation to formulate relevant home-based rehabilitation interventions may be an effective way to improve the QoL of type B AD survivors.

The strengths of our study are that it is the first survey to identify health promotion behavior status in patients with type B AD. Moreover, we assessed QoL in patients with type B AD and validated the associated risk factors. However, this study had some limitations. First, participant selection was performed by convenience sampling from only one hospital in China. Second, participants used self-report measures, thus their answers are subject to reporting bias. Third, the cross-sectional nature of the study hinders our ability to make causal inferences regarding risk factors and diseases that exist concurrently.

In conclusion, improving the motivation of health promoting behavior may be an effective measure to improve the QoL of patients.

## Data Availability

Data shall only be shared with researchers upon reasonable request, at the discretion of the principal investigator.
